# Developing measures on the perceptions of the built environment for physical activity: a confirmatory analysis

**DOI:** 10.1186/1479-5868-7-72

**Published:** 2010-10-07

**Authors:** Jennifer L Gay, Kelly R Evenson, Jessalyn Smith

**Affiliations:** 1 Division of Behavioral Sciences and Health Promotion, University of Texas School of Public Health, Brownsville, TX, USA; 2 Department of Epidemiology, Gillings School of Global Public Health, University of North Carolina, Chapel Hill, North Carolina, USA; 3 CTB/McGraw Hill, Monterey, CA, USA

## Abstract

**Background:**

Minimal validity evidence exists for scales assessing the built environment for physical activity. The purpose of this study was to assess the test-retest reliability and invariance of a three-factor model (Neighborhood Characteristics, Safety/Crime, and Access to Physical Activity Facilities) across gender, race, geographic location, and level of physical activity.

**Methods:**

To assess measurement invariance, a random sample of 1,534 adults living in North Carolina or Mississippi completed a computer assisted telephone interview that included items examining perceptions of the neighborhood for physical activity. Construct level test-retest reliability data were collected from a purposeful sample of 106 participants who were administered the questionnaire twice, approximately two weeks apart. Fit indices, Cronbach's alpha, Mokken H and Spearman correlation coefficients (SCC) were used to evaluate configural and co/variance invarianc,e and intraclass correlation coefficients (ICC) were used to assess reliability.

**Results:**

Construct test-retest reliability was strong (ICC 0.90 to 0.93). SCC for Neighborhood Characteristics and Crime/Safety were weak with Access (0.21 and 0.25), but strong between Crime/Safety and Neighborhood Characteristics (0.62). Acceptable fit and evidence of measurement invariance was found for gender, race (African American and White), geographic location, and level of physical activity. Fit indices consistently approached or were greater than 0.90 for goodness of fit index, normed fit index, and comparative fit index which is evidence of configural invariance. There was weak support of variance and covariance invariance for all groups that was indicative of factorial validity.

**Conclusions:**

Support of the validity and reliability of the three-factor model across groups expands the possibilities for analysis to include latent variable modeling, and suggests these built environment constructs may be used in other settings and populations.

## Background

With the advent of ecological models, physical activity research now frequently incorporates built environment measures [1]. While there is a clear cross-sectional association between built environmental characteristics and physical activity, the majority of research is conducted at the item level [2]. Analysis of individual items ignores the potential underlying themes or constructs that may exist, particularly in perceptual measures. Further, item-level analysis precludes the use of multilevel modeling techniques that can account for the latent constructs inherent in measures of beliefs and attitudes [3].

Several scales exist that measure perceptions of the built environment for physical activity among adults [4-7]. However, little evidence is available regarding the validity or reliability of these measures. The most commonly reported measurement property is test-retest reliability [2]. To date few studies report the construct validity, including factorial validity, of perceptions of the built environment for physical activity. Construct validity is necessary for operationalizing variables and making inferences. Factorial validity is a type of construct validity that applies to the structure of how latent, or underlying, constructs are measured using scales of multiple items. Each item on a scale should strongly relate to one latent construct and weakly relate to any other constructs being measured [8].

In 2005, Evenson and McGinn [6] developed a questionnaire for adults examining perceptions of the built environment for physical activity using the framework of Pikora et al [9] for perceptions around walking and cycling. The framework included the following physical environmental domains: destination, functionality, aesthetic, and safety. The destination feature relates to the availability of public and private facilities. The functionality feature reflects the physical attributes of the street and path that make up the fundamental structural aspects of the local environment, such as the type and width of the street and the volume, speed, and type of traffic. The aesthetic feature included both streetscape (e.g., trees, garden and street maintenance, cleanliness, pollution) and views (e.g., sights, architecture). The safety feature represents both personal safety and traffic safety. Item-level test-retest reliability was between 0.4 and 0.8 (intraclass correlation coefficients) among a sample of African American and White adults [6].

A recent examination of the psychometric properties of 26 items from this questionnaire in a separate sample of 479 White and African American adults, along with 21 items regarding convenience of physical activity facilities from Sallis et al. [10], revealed a five-factor structure [11] different than the Pikora et al [9] framework. The Convenience items formed one factor, while 16 items from the Evenson and McGinn [6] questionnaire produced four factors: Crime/Safety, Neighborhood Characteristics, Access to Physical Activity Facilities (referred to as Access), and Places of Worship [11]. The internal consistency and scalability coefficients of these constructs indicated separate constructs. However, the sample size in this study and the relative homogeneity of the sample in terms of gender (86% female), race (68% White), 100% from four urban areas in South Carolina, and level of exercise (92% did not meet physical activity recommendations) [12] precluded further testing of construct validity. Measurement invariance means that the same latent construct is being measured across groups. If a measure is invariant by group membership there is evidence that the measure is not biased, and allows for mean comparisons of the latent constructs. Confirming the factor structure and testing the measurement invariance are the next steps in establishing validity and reliability for the new factor structure described in Gay and Smith [11].

Using self-reported built environment data collected on a diverse sample of adults, this paper had two aims: 1) to confirm the factor structure, reliability, and scalability of three of the five factors (Neighborhood Characteristics, Crime/Safety, and Access) found in Gay and Smith [11] by gender, race/ethnicity, physical activity level, and geographic location, and 2) to assess the test-retest reliability of these constructs. The Convenience and Places of Worship factors from the prior study were not tested since the confirmatory data did not contain the requisite items.

## Methods

### Sample

A telephone survey was conducted using a computer assisted telephone interview system (CATI) between January and July 2003 on a random sample of non-institutionalized adults 18 years or older residing in two regions: Forsyth County, North Carolina and the metropolitan statistical area (MSA) of Jackson, Mississippi. Disproportionate sampling was used for Forsyth County in order to ensure representation for less urban areas outside of the Winston-Salem metropolitan area within the county. Respondents were randomly chosen in two stages: the first stage at the household level and the second stage at the individual level. Surveys were only conducted in English. Each participant provided consent and the study was approved by the Institutional Review Board at the University of North Carolina. Participants were paid $5 for their participation for each survey they completed. More detail is available elsewhere [6].

### Reliability Interviews

Overall 1,662 men and women completed the survey. At the end of the interview, 1,448 adults were asked if they would be willing to participate in a retest interview. The remaining 214 adults were not asked to participate in a retest interview, because the interview quota was complete. Among these 1,448 adults, 76% (n = 1,104) agreed to be called back for the retest survey. Reliability information was collected from a 6% (n = 106) purposeful sample of women and men, to ensure approximately equal numbers of participants from both sites, by gender, and by race/ethnicity. The mean time between interviews was 16.8 days (standard deviation 4.2, range 9-30 days).

### Questionnaire

#### Physical Activity

Physical activity was assessed by asking if the adults had participated in any moderate- or vigorous-intensity activity for at least 10 minutes at a time, using questions from the year 2001 BRFSS core module on physical activity [13]. If they responded "yes" to either question on moderate- or vigorous-intensity activity, then they were asked on how many days per week did they engage in the activity for at least 10 minutes at a time, and how much total time per day they spent doing these activities. We grouped participants into two groups based on current physical activity guidelines [14]: Meeting guidelines was calculated as moderate-intensity activity for at least 150 minutes per week, or vigorous-intensity activity for at least 75 minutes per week, or a combination of the two (treating vigorous activity as twice as many minutes as moderate-intensity activity) [15]. If participants did not report enough activity to meet guidelines they were classified as Not Meeting Guidelines.

#### Other Measures

All respondents were asked questions regarding age, gender, race/ethnicity, marital status, education, and employment. Employment was grouped into two categories: employed or not employed (out of work, homemaker, student, retired, or unable to work). Self-reported height and weight were collected to calculate body mass index (BMI).

### Analysis Plan

The analysis includes three factors from the exploratory analysis presented in Gay and Smith [11]: Neighborhood Characteristics, Crime/Safety, and Access. Thirteen items (Additional file: 1) were included in the confirmatory factor analysis (CFA). Cronbach's alpha [16] was calculated to assess internal consistency of the three factors, and values greater than 0.70 were considered acceptable. Mokken *H*, a measure of scale homogeneity, was also used to verify the consistency of the three factors. An *H *between 0.30 and 0.40 denoted a weak scale, 0.40 to .50 a moderate scale, and 0.50 to 1.00 a strong scale [17].

Intraclass correlation coefficients were calculated to examine the test-retest reliability of the three factors. Landis and Koch [18] suggest that agreement values are slight or poor if less than or equal to 0.20, 0.21 to 0.40 is fair, 0.41 to 0.60 is moderate, 0.61 to 0.80 is substantial, and almost perfect is greater than 0.80. Separate invariance tests were conducted by level of activity, gender, race/ethnicity, and geographic location. Geographic location was defined as Jackson, Mississippi, Winston-Salem, North Carolina, and Forsyth County, North Carolina, where the Forsyth County sample refers to all areas except Winston-Salem; those areas were both suburban and rural. Mokken scaling was conducted in R [19]. All other analyses were conducted using LISREL [20].

#### Statistics Used to Determine Measurement Invariance

Measurement model fit holds if the goodness-of-fit index (GFI), normed fit index (NFI), and comparative fit index (CFI) are >0.90. The lower cutoff of 0.90 is used because this is not a well-established instrument that is in the formative stage. We also examine the standardized root-mean-square residual (SRMR; values <0.05), and the root-mean-square error of approximation (RMSEA; values less than or equal to 0.08 indicated acceptable fit) and the expected cross-validation index (ECVI; values closer to zero).

Measurement invariance holds if the constraints make a significant improvement in the model fit. Typically, to assess this, the Δχ^2 ^is examined between two nested models. This value follows a χ^2 ^distribution with the degrees of freedom equal to the difference of the degrees of freedom between the nested models. If measurement invariance holds, there will be a non-significant improvement in fit. However, some have questioned the usefulness of the Δχ^2 ^[21,22] since it is a function of the sample size. Therefore, Δχ^2 ^may reject trivial differences in the model that do not have much practical importance. As a result, some practitioners recommend using the change of fit indices to determine whether measurement invariance holds. Hu and Bentler [23] recommend ΔCFI, if it is within 0.01, indicating evidence that measurement invariance holds. This is the criterion we used to assess measurement invariance.

#### Types of Measurement Invariance

Configural invariance is tested to determine whether the conceptual framework is the same across different groups [24-26]. Here the pattern of the free and fixed loadings is the same across groups. Lack of evidence of configural invariance indicates that measurement invariance does not hold. Therefore, no further invariance testing should be done [24-27]. Factor co/variance invariance is tested to determine if the variance covariance structure across groups holds. If both the factor variances and covariances are invariant, the correlations between the constructs are invariant as well. If error variances are invariant across groups, this indicates that the measurement error is invariant across groups. If it is found that measurement invariance holds, the items can be assumed to be equally reliable across groups.

## Results

The sample consisted of 1,534 adults (mean age = 47.88 ± 17.05) living in Jackson, Mississippi (n = 741), Forsyth County, Winston-Salem, North Carolina (n = 379), and Forsyth County, North Carolina rural areas (n = 414). Nearly two-thirds of the sample was female (66.8%), 91.2% graduated high school and 42.6% attended at least 4 years of college. Just over half (61.7%) of participants were employed. Less than half of the sample was married (45.7%) with the next largest group being those who were never married (20.4%). More than one-third (36.3%) of the sample was Black, non-Hispanic while the majority were White, non-Hispanic (58.8%). The mean BMI for the sample was 27.2 kg/m ^2 ^(SD = 6.26) and 61.5% of participants met physical activity guidelines (150 minutes or more of moderate-intensity physical activity, 75 minutes of vigorous activity, or a combination of moderate- and vigorous-intensity activity per week).

Means, standard deviations, and ranges for the three constructs are provided in Table 1. Sum score means are also provided as these constructs may be treated as indices. The Neighborhood Characteristics construct had acceptable internal consistency. The Crime/Safety and Access constructs had adequate internal consistency coefficients above 0.60. The Mokken *H *scalability coefficient was strongest for Neighborhood Characteristics (0.61) indicating a strong scale. Crime/Safety had a moderate scalability coefficient, and the *H *for Access was weak.

**Table 1:** Sample Means (M), Standard Deviations (SD), Sum Score Means, Cronbach's α, Mokken *H*, Intraclass Correlation Coefficients (ICC) with 95% Confidence Intervals (CI), and Spearman Rho Correlation Coefficients for the Three Factor Model (N = 1,534)

	Sample Means		Sample Sum Score Means					Spearman Rho		
**Factor**	*M*	*SD*	*M*	*SD*	**α**	***H***	**ICC (95% CI)**	**1.**	**2.**	**3.**

1. Neighborhood Characteristics	2.28	0.61	9.06	2.44	0.80	0.61	0.93 (0.90,0.95)	1.00		
2. Crime/Safety	1.99	0.54	11.84	3.27	0.67	0.49	0.93 (0.90,0.95)	0.62*	1.00	
3. Access	3.87	0.75	10.71	2.69	0.64	0.34	0.90 (0.86,0.93)	0.21*	0.25*	1.00

*p < .0001

Note: Ranges for the Sample Means were 1.00 to 4.00 for each of the constructs. For the Sum Score Means the range was 3.00 to 16.00 for Neighborhood Characteristics, 2.00 to 24.00 for Crime/Safety, and 1.00 to 15.00 for Access. All Items were coded such that higher scores indicated a more favorable perception of the environment.

Construct test-retest reliability was assessed using intraclass correlations (Table 1). All three constructs had high ICCs indicating almost perfect test-retest reliability [18]. There was a strong, positive correlation between Neighborhood Characteristics and Crime/Safety, and weak positive associations with Access (all items were coded so that higher scores indicated a more favorable perception of the environment).

### Group Invariance - Gender

Measurement models for all groups produced acceptable factor loadings. Table 2 provides model fit indices for each group in the invariance testing. For the GFI, NFI, and CFI, values above 0.90 indicate strong fit [28,29]. Since the model values were slightly lower than 0.90, there was evidence of fair model fit. Additionally, SRMR is only slightly above the 0.05 cutoff, in support of acceptable fit. The RMSEA was larger than ideal (0.05) cutoff, indicating less support of acceptable fit. With the exception of the GFI, the models have the same degree of fit across gender. ΔCFI (Table 3) indicated that there was some evidence of configural measurement invariance. However theΔ χ^2 ^indicated a lack of configural measurement invariance. Additionally, both the Δχ^2 ^and ΔCFI indicated the factor variances and covariances between factors were the same for both males and females. ΔCFI indicated that there was some evidence of error variances being equivalent for both males and females. As a result, since only the ΔCFI favored measurement invariance, there was evidence of weak measurement invariance for males and females.

**Table 2:** Measurement Model Fit for Gender, Activity Level, Race/Ethnicity, and Geographic Location Invariance Tests

	**χ**^**2**^	GFI	SRMR	NFI	CFI	RMSEA	ECVI	Critical N
Gender								
Male	319.70	0.76	0.08	0.87	0.89	0.11	1.02	104.29
Female	647.54	0.87	0.08	0.86	0.87	0.12	1.08	91.87
								
Meeting Guidelines for Physical Activity								
Not Meeting Guidelines	363.14	0.91	0.07	0.90	0.91	0.10	0.75	138.96
Meeting Guidelines	500.57	0.91	0.08	0.89	0.90	0.09	0.68	147.42
								
Race/Ethnicity								
White	325.66	0.89	0.09	0.86	0.87	0.11	0.85	116.66
Black	817.78	0.92	0.07	0.90	0.90	0.09	0.65	150.53
								
Geographic Location								
Forsyth County, North Carolina - suburban/rural	294.92	0.89	0.08	0.86	0.88	0.10	0.93	116.72
Jackson, MS	380.09	0.92	0.06	0.90	0.91	0.09	0.67	157.75
Winston-Salem, North Carolina - urban	283.95	0.90	0.09	0.87	0.90	0.10	0.91	121.30

GFI - Goodness-of-fit index; SRMR - Standardized root mean square residual; NFI - Normed fit index; CFI - Comparative fit index; RMSEA - Root mean square error of approximation; ECVI - Expected cross-validation index

**Table 3:** Gender Invariance Testing

	***χ***^**2**^	*ν*	CFI	NNFI	ECVI	Δ*ν*	**Δ*χ***^**2**^	p-value	Δ*CFI*
Baseline	927.238	124	0.877	0.845	1.056				
Factor loadings	979.258	134	0.877	0.857	1.049	10	52.020	<0.001	0.000
Var/cov	987.478	140	0.876	0.862	1.046	6	8.220	0.220	0.001
Errors	1034.223	153	0.870	0.867	1.081	13	46.745	<0.001	0.006

CFI - Comparative fit index; NNFI - Normed fit index; ECVI - Expected cross-validation index

### Group Invariance - Meeting Guidelines for Physical Activity

Based on the model fit information (Table 2), there was no indication that there was a lack of model fit. Both the group meeting physical activity guidelines and those not meeting guidelines had nearly the same model fit. For both groups, the SRMR and RMSEA were higher than the 0.08 cutoff. The results indicate that there was weak measurement invariance across activity levels since there was no change in the CFI (Table 4).

**Table 4:** Meeting Guidelines for Physical Activity Invariance Testing

	***χ***^**2**^	*ν*	CFI	NNFI	ECVI	Δ*ν*	**Δ*χ***^**2**^	p-value	Δ*CFI*
Baseline	863.666	124	0.904	0.879	0.712				
Factor loadings	907.756	134	0.900	0.884	0.727	10	44.09	<0.001	0.004
Var/cov	921.734	140	0.899	0.887	0.732	6	13.978	0.0299	0.001
Errors	953.803	153	0.896	0.894	0.737	13	32.069	0.0023	0.003

CFI - Comparative fit index; NNFI - Normed fit index; ECVI - Expected cross-validation index

### Group Invariance - Race/Ethnicity

As the GFI, NFI, and CFI were all close or above 0.90 (Table 2) there was strong indication that the measurement model fit for non-Hispanic Black individuals. However, for those identifying as non-Hispanic White, the model fit indices were lower than the values indicative of acceptable fit. The results (Table 5) indicate that there was weak measurement invariance across race since there was a slight change in the CFI. However, the Δχ^2 ^and the ΔCFI support measurement invariance across the loadings.

**Table 5:** Race/Ethnicity Invariance Testing

	***χ***^**2**^	*ν*	CFI	NNFI	ECVI	Δ*ν*	**Δ*χ***^**2**^	p-value	Δ*CFI*
Baseline	1443.443	124	0.891	0.862	0.721				
Factor loadings	1454.702	134	0.890	0.872	0.718	10	11.259	0.338	0.001
Var/cov	1463.356	140	0.890	0.877	0.717	6	8.645	0.195	0.000
Errors	1506.707	153	0.889	0.886	0.720	13	43.351	<0.001	0.001

CFI - Comparative fit index; NNFI - Normed fit index; ECVI - Expected cross-validation index

### Group Invariance - Location

Model fit for Jackson, Mississippi, Winston-Salem, North Carolina urban, and Forsyth County, North Carolina (suburban/rural) is shown in Table 2. The model fit for individuals from Jackson, Mississippi was slightly better than for those from Forsyth County, North Carolina and Winston-Salem, North Carolina. There was an indication that the measurement model fit for these locations since for all locations the GFI, NFI, and CFI met or approached 0.90. The RMSEA was higher than desirable for all locations. The results indicated that there was weak measurement invariance across all locations (Table 6) as the p-values were small and the ΔCFI was small. With the slight change in the ΔCFI for factor loadings, the locations exhibited some configural invariance.

**Table 6:** Invariance Testing by Geographic Location (Jackson, Mississippi; Winston-Salem, North Carolina urban; and Forsyth County, North Carolina (suburban/rural; excluding Winston Salem)

	***χ***^**2**^	*ν*	CFI	NNFI	ECVI	Δ*ν*	**Δ*χ***^**2**^	p-value	Δ*CFI*
Baseline	958.955	186	0.900	0.874	0.792				
Factor loadings	1001.407	206	0.897	0.883	0.796	20	42.452	0.002	0.003
Var/cov	1039.906	218	0.892	0.884	0.814	12	38.499	<0.001	0.005
Errors	1166.810	244	0.882	0.887	0.852	26	126.903	<0.001	0.01

CFI - Comparative fit index; NNFI - Normed fit index; ECVI - Expected cross-validation index

## Discussion

Measuring perceptions of the built environment for physical activity has become more prevalent as the use of ecologic models increases in the physical activity domain [1]. Missing from much of the built environment literature are validity tests of the self-report instruments. The purpose of this paper was the test the factor structure, reliability, and scalability of the three factors (Neighborhood Characteristics, Crime/Safety, and Access) found in Gay and Smith [11] using a larger confirmatory sample from Evenson and McGinn [6]; we also examined the factorial validity of the constructs by level of physical activity, gender, race/ethnicity, and geographic location using tests of configural invariance.

The means, standard deviations, and ranges for the Neighborhood Characteristics and Crime scales were similar to the values found in Gay and Smith [11], but the mean value for Access to physical activity facilities was higher in the overall sample for this study (3.87 ± 0.75) than in the exploratory study (2.16 ± 0.58). Regardless, the measurement model fit was acceptable in this study. Similarly the scales exhibited adequate reliabilities for both internal consistency and test-retest reliability. The Mokken *H *scalability coefficients were slightly higher in this study for Neighborhood Characteristics and lower for Crime, but still moderate-to-strong for both scales. The Neighborhood Characteristics scale is similar to the Neighborhood Environment Walkability Scale (NEWS) Traffic Hazards subscale identified in the Baltimore, Maryland [30] and Australian samples [4]. The Crime scales from this study and from the NEWS studies contain many of the same items. The Access scale did not align with items from NEWS. While two of the three scales are similar, the NEWS focuses solely on walking behavior. The current study includes all forms of physical activity in the neighborhood. The differences in factor structures of this study and the NEWS may reflect perceptual variations based on type of activity.

The configural invariance was tested to examine the theoretical framework across gender, race/ethnicity, physical activity group, and geographic location as the participants came from three distinct areas. There was weak measurement invariance for all group invariance tests and indications that the measurement model had acceptable fit based on the GFI, NFI and CFI. The RMSEA, generally less affected by large sample sizes, was larger than expected. However, the spectrum of fit indices indicated acceptable fit across all groups. The factor structures were the same as the a priori model resulting from the exploratory factor analysis [11]. While the evidence is not as strong as desired, there is sufficient confirmation of the factors to conduct further validation studies using these scales. Future research may consider further instrument development and testing of the psychometric properties.

This study is unique as we have provided initial support for the generalizability of the factor structure for perceptions of the built environment for physical activity across race/ethnicity, gender, level of activity, and perhaps most importantly geographic location. Given that the built environment, and therefore perceptions, can change by neighborhoods, cities, or rural or urban location, validity of the factor structure across geographically diverse areas encouraging. One possible implication of these findings is that this scale can be used to assess perceptions in various settings. As changing perceptions of the built environment may increase physical activity, these factors may be used to determine targets for built environmental change.

### Limitations

The findings from this study should be taken within the context of several limitations. First, neither this sample nor the original exploratory paper had samples that included a large proportion of Hispanics or other races such as Asian or Native American. The survey and factor structure should be tested in more diverse populations and in other languages. Second, the version of the measure used in this study did not include the Convenience or Places of Worship scales [10]. Therefore the Convenience and Places of Worship factors from the exploratory study [11] could not be tested. Finally, participants were asked to consider their neighborhood as the area within a 20-minute walk or one-mile from their home. While the purpose of the study and the built environment items was to capture physical activity near their home, participants may engage in physical activity in areas outside of these boundaries and indeed the measure of physical activity was more general. Our results may have been stronger for physical activity if we focused on physical activity also conducted within one mile of their home, as there may have been a disconnect between the perceptions of the neighborhood for activity and the amount of physical activity if the person is active outside of the neighborhood [31].

## Conclusions

This research contributes to the evidence by providing additional support for the factor structure of a survey measuring the perceptions of the built environment for physical activity. Currently the evidence lacks appropriate examinations of these items and subscales not only across populations, but also settings, particularly as the NEWS focuses on built environmental attributes for walking, not physical activity more broadly. We have explored the factorial structure and results indicate that the subscales apply to suburban/rural and urban settings, across race/ethnicity, gender, and whether or not physical activity guidelines were met. Having a generalizable factor structure expands the possible analyses beyond item-level variables and allows for the creation of factor scores for use in statistical analysis as well as in latent variable modeling. Using such thematic or latent analyses may allow for targeting specifics of groups of environmental characteristics that most impact physical activity. These strategies are used frequently in psychology and education domains, from which public health draws. The results from this study contribute to establishing validity for a perceptual measure of the built environment for physical activity. Furthering the measurement of perceptions of the built environment may contribute to improved interventions and ultimately increased physical activity.

## Competing interests

The authors declare that they have no competing interests.

## Authors' contributions

JLG conceptualized the manuscript, drafted the manuscript and conducted some statistical analyses. KRE designed the questionnaire, conducted the project from which the data are derived, and provided feedback on drafts of the manuscript. JS provided input on and conducted some statistical analyses, and provided feedback on drafts of the manuscript. All authors read and approved the final manuscript.

## Supplementary Material

Additional file 1 **Items, by factor, included for invariance testing**. Click here for file
