# Supplementary material for: Developing measures on the perceptions of the built environment for physical activity: a confirmatory analysis
Source: Int J Behav Nutr Phys Act. 2010 Oct 7;7:72. doi: 10.1186/1479-5868-7-72 (PMC2959084; doi:10.1186/1479-5868-7-72)
Supplement: Additional file 1 — Items, by factor, included for invariance testing. [file 1479-5868-7-72-S1.DOCX]

Appendix 1. Items, by factor, included for invariance testing

| Neighborhood Characteristics | |
| --- | --- |
|  | Heavy traffic is a problem in my neighborhood. |
|  | Speeding cars are a problem in my neighborhood. |
|  | Lack of crosswalks or traffic signals to help cross streets is a problem in my neighborhood. |
|  | Unattended dogs are a problem in my neighborhood. |
| Crime/Safety | |
|  | There is a high crime rate in my neighborhood. |
|  | The crime rate in my neighborhood makes it unsafe to go on walks during the day.. |
|  | The crime rate in my neighborhood makes it unsafe to go on walks at night.. |
|  | Trash, litter or graffiti is a problem in my neighborhood. |
|  | Exhaust fumes or other pollution is a problem in my neighborhood. |
|  | Lack of trees along the street that provide shade is a problem in my neighborhood. |
| Access to Physical Activity Facilities | |
|  | Would you say the availability of public recreational facilities in your community was… |
|  | Would you say the availability of private recreational facilities in your community was… |
|  | Would you say the availability of facilities at public schools in your community was… |

For Neighborhood Characteristics and Crime/Safety items, 1 = “Strongly Agree” and 4 = “Strongly Disagree”. The three Access items had a response format of 1 = “Excellent” and 4 = “Poor”.
